# Elucidating the genetic architecture of reproductive ageing in the Japanese population

**DOI:** 10.1038/s41467-018-04398-z

**Published:** 2018-05-17

**Authors:** Momoko Horikoshi, Felix R. Day, Masato Akiyama, Makoto Hirata, Yoichiro Kamatani, Koichi Matsuda, Kazuyoshi Ishigaki, Masahiro Kanai, Hollis Wright, Carlos A. Toro, Sergio R. Ojeda, Alejandro Lomniczi, Michiaki Kubo, Ken K. Ong, John. R. B. Perry

**Affiliations:** 1 Laboratory for Endocrinology, Metabolism and Kidney Diseases, RIKEN Centre for Integrative Medical Sciences, Yokohama, 230-0045 Japan; 2 MRC Epidemiology Unit, University of Cambridge School of Clinical Medicine, Institute of Metabolic Science, Cambridge Biomedical Campus, Cambridge, CB2 0QQ UK; 3 Laboratory for Statistical Analysis, RIKEN Center for Integrative Medical Sciences, Yokohama, 230-0045 Japan; 4 Laboratory of Genome Technology, Human Genome Center, Institute of Medical Science, The University of Tokyo, Tokyo, 108-8639 Japan; 5 Center for Genomic Medicine, Kyoto University Graduate School of Medicine, Kyoto, 606-8507 Japan; 6 Department of Biomedical Informatics, Harvard Medical School, Boston, MA 02115 USA; 7 Primate Genetics Section/Division of Neuroscience, Oregon National Primate Research Center, Oregon Health and Sciences University, Beaverton, OR 97006 USA; 8 Division of Neuroscience, Oregon National Primate Research Center, Oregon Health and Sciences University, Beaverton, OR 97006 USA; 9 RIKEN Center for Integrative Medical Sciences, Yokohama, 230-0045 Japan

## Abstract

Population studies elucidating the genetic architecture of reproductive ageing have been largely limited to European ancestries, restricting the generalizability of the findings and overlooking possible key genes poorly captured by common European genetic variation. Here, we report 26 loci (all *P* < 5 × 10^–8^) for reproductive ageing, i.e. puberty timing or age at menopause, in a non-European population (up to 67,029 women of Japanese ancestry). Highlighted genes for menopause include *GNRH1*, which supports a primary, rather than passive, role for hypothalamic-pituitary GnRH signalling in the timing of menopause. For puberty timing, we demonstrate an aetiological role for receptor-like protein tyrosine phosphatases by combining evidence across population genetics and pre- and peri-pubertal changes in hypothalamic gene expression in rodent and primate models. Furthermore, our findings demonstrate widespread differences in allele frequencies and effect estimates between Japanese and European associated variants, highlighting the benefits and challenges of large-scale trans-ethnic approaches.

## Abstract (web summary)

The timing of female reproductive capacity is influenced by genetic and environmental factors. Here, in genome-wide association studies, the authors identify genetic loci for age at menarche and onset of menopause in Japanese women, and highlight differences with European populations.

## Introduction

The first menstrual period (‘menarche’) and onset of menopause are key milestones of female reproductive ageing, representing the start and end of reproductive capacity. The timings of these events vary widely between individuals and are predictors of the rate of ageing in other body systems^1^. This variation reflects a complex mix of genetic and environmental factors that population-based studies are beginning to unravel. Over the past decade, successive waves of genome-wide association study (GWAS) meta-analyses have illuminated the genetic architecture of reproductive ageing and shed light on several underlying biological processes, many of which are also highlighted through studies of rare human disorders of reproduction^2–7^. Hence, puberty timing appears to be predominantly regulated by the central nervous system, including components of the hypothalamic–pituitary axis and complex molecular silencers of that system^5^. In contrast, the aetiological drivers of menopause timing appear to be ovary-centric, largely focussed on the ability of oocytes to maintain genome stability and hence preserve the ovarian primordial follicle pool^7^.

A key limitation of previous GWAS for reproductive ageing is their large circumscription to populations of European ancestry, due to the lack of available large-scale studies of other populations. This population restriction has limited the generalizability of the findings and may have led to a failure to detect key genes and pathways that are poorly represented by common functional variants in European populations. Previous small-scale genetic studies in East Asian and African–American samples have replicated a small number of the reported European loci^8–10^. However, such studies have not yet demonstrated any known or new genetic association signals for menarche or menopause timing at genome-wide statistical significance. To address this limitation, here we describe two separate GWAS for ages at menarche and menopause in up to 67,029 women of Japanese ancestry from the BioBank Japan (BBJ) Project^11^. This sample represents a threefold larger sample size than any previous non-European ancestry GWAS^9^. We identify 26 loci for ages at menarche or menopause at genome-wide significance, implicating several new genes and pathways. The analyses also reveal widespread differences in effect estimates between populations, highlighting the benefits and challenges of trans-ethnic GWAS meta-analyses.

## Results

### Effects of previously reported European loci in Japanese

Data on age at natural menopause and age at menarche were available for 43,861 and 67,029 genotyped women of Japanese ancestry, respectively (Supplementary Table 1). Genotyping array-based heritability in this Japanese sample was 10.4% (S.E. 0.9%) for menopause (contrasting with 36% in Europeans) and 13% (S.E. 0.6%) for menarche (32% in Europeans). Mean age at menarche in this Japanese population (overall: 13.9 years) was higher than previously reported in contemporary European populations (12.4–13.7 years)^5^, but showed a marked secular trend in Japanese from 15.2 years in women born pre-1935 to 12.3 years in those born post-1965, and this was accompanied by increasing heritability (from 14.2% to 20.6%; *P*_het_ = 0.03, Supplementary Table 2).

Of the 54 previously identified European menopause loci (Supplementary Data 1), 52 were polymorphic in Japanese; of these 46 (88.4%) had a consistent direction of effect (binomial *P* = 1 × 10^−8^; 29 loci at nominal significance *P* < 0.05). For menarche, 348/377 autosomal variants found in Europeans were present in the Japanese dataset (Supplementary Data 2); of these 282/348 (81.0%) had a consistent direction of effect (binomial *P* = 6.4 × 10^−33^, 108 loci at *P* < 0.05). In aggregate, genetic variation ± 250 kb from European-identified SNPs explained 2% (S.E. 0.2%) and 3.6% (S.E. 0.2%) of the trait variance for age at menopause and menarche, respectively (contrasting with 8.0% [S.E. 0.5%] and 8.4% [S.E. 0.4%] in Europeans, respectively).

There were notable differences in allele frequencies between populations at these European-identified signals (Supplementary Data 1), with 23 loci (2 menopause, 21 menarche) monomorphic in Japanese. The mean absolute difference in allele frequency was 17%, with the largest difference at the menarche locus, 20q11.21, where the C-allele at rs1737894 in Europeans (frequency ~60%) is absent in Japanese.

To compare effect sizes between populations at these previously identified signals, we calculated effect estimates for Europeans in up to 73,397 women from the UK Biobank study, independent of the European discovery samples. Across 52 (polymorphic in Japanese) menopause loci, 44 (84.6%) showed a larger effect in Europeans than in Japanese (binomial *P* = 4 × 10^−7^), 23 of which were significantly different (*P*_diff_ < 0.05, Supplementary Data 1). Similarly, for the 102 menarche loci that were previously identified in Europeans excluding UK Biobank (Supplementary Data 3), 77 (75.5%) showed larger effects in UK Biobank Europeans than in Japanese (binomial *P* = 2.5 × 10^−7^, 22 at *P*_diff_ < 0.05). We were able to compare our Japanese allele frequencies and effect estimates to Chinese data for a subset of known European loci from a recent study^10^. For both menarche and menopause there was high concordance in both allele frequency (minimum *r*^2^ = 0.95, Supplementary Figure 1) and effect estimates (*r*^2^ = 0.5, Supplementary Figure 2).

In aggregate, our findings likely indicate widespread population differences in LD between GWAS signals and the underlying causal variants, or possible differences in modifying environmental factors.

### Menopause and menarche signals in Japanese

**Fig. 1:**
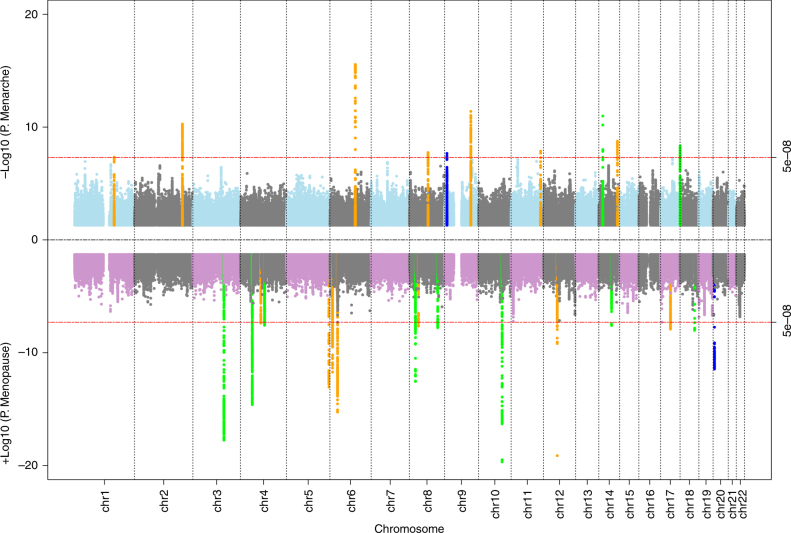
‘Miami’ plot showing genome-wide association test statistics for ages at menarche (top) and menopause (bottom) in the BioBank Japan Project. Genome-wide significant loci in BBJ are highlighted according to: novel loci (green), novel signals at known loci (blue) and known loci (orange)

To identify genetic signals for ages at menopause and menarche in our Japanese population, we tested genome-wide markers imputed to the 1000 genomes Phase 3 reference panel^12^. For menopause, 16 independent signals reached genome-wide significance (*P* < 5 × 10^−8^), 8 of which are novel and not previously reported in Europeans (Table 1; Fig. 1). Additionally, we found a signal (rs76498344, *P*_Japanese_ = 3.6 × 10^−12^) near the previously reported locus *MCM8* which was uncorrelated with the reported European lead SNP (rs451417, *P*_Japanese_ = 0.002, *r*^2^ = 0.03 with rs76498344 in Japanese) and showed no association in Europeans (rs76498344 *P*_Euro_ = 0.78)^5^. Formal conditional analysis confirmed the independence of these two signals (*P*_unadjusted_ = 3.6 × 10^−12^, beta_unadjusted_ = 0.22 *P*_adjusted for rs451417_ = 1.7 × 10^−10^, beta_adjusted_ = 0.20).

**Table 1:** Genome-wide significant signals identified for ages at menarche and menopause in the BioBank Japan Project

Location	SNP (*r*^2^)^a^	Alleles^b^	Nearest Gene	Japanese (BBJ)		European samples		Het. *P*
				Effect (S.E)	*P*	Effect (S.E)	*P*	
Novel signals at novel loci								
Menopause								
3q21.3	rs4853	T/C/0.94/0.90	*H1FX*	0.48 (0.05)	2E−18	0.03 (0.05)	5E−01	2E−09
4p11	rs10049761	T/G/0.36/0.50	*ZAR1*	0.22 (0.03)	2E−15	0.08 (0.03)	4E−03	1E−03
4q23	rs199646819	A/ATGG/0.61/0.02	*EIF4E*	0.15 (0.03)	3E−08	0.19 (0.11)	7E−02	7E−01
8p21.2	rs6185	G/C/0.51/0.25	*GNRH1*	0.19 (0.03)	3E−13	0.04 (0.04)	3E−01	7E−04
8q24.11	rs2921759	T/C/0.82/0.98	*RAD21*	0.19 (0.03)	2E−08	−0.11 (0.12)	4E−01	1E−02
10q24.1	rs1889921	T/G/0.53/0.53	*CCNJ*	0.24 (0.03)	2E−20	0.11 (0.03)	1E−04	7E−04
14q24.2	rs8010674	C/T/0.63/0.62	*DCAF4*	0.15 (0.03)	3E−08	0.07 (0.03)	3E−02	5E−02
18q21.33	rs200296776	C/T/0.99/0.999	*ZCCHC2*	1.15 (0.20)	9E−09	−0.63 (1.69)	6E−01	3E−01
Menarche								
14q13.3	rs2076751	C/A/0.75/0.93	*NKX2*-*1*	0.07 (0.01)	1E−11	0.07 (0.02)	3E−05	9E−01
18p11.32	rs77001758	A/G/0.40/0.001	*THOC1*	0.05 (0.01)	5E−09	0.32 (0.15)	4E−02	8E−02
Novel signals at known loci								
Menopause								
20p12.3	rs76498344 (0.03)	C/T/0.23/0.05	*MCM8*	0.22 (0.03)	4E−12	0.01 (0.07)	8E−01	7E−03
Menarche								
9p23	rs291269 (0)	G/A/0.37/0.32	*PTPRD*	0.05 (0.01)	2E−08	0.01 (0.01)	3E−01	9E−04
Genome-wide significant signals correlated with known European signals								
Menopause								
4q21.23	rs7665103 (0.97)	G/A/0.62/0.41	*HELQ*	0.15 (0.03)	4E−08	0.25 (0.03)	5E−17	1E−02
5q35.2	rs34933909 (0.91)	T/G/0.49/0.45	*UIMC1*	0.20 (0.03)	9E−14	0.32 (0.03)	2E−26	4E−03
6p24.2	rs12211124 (0.22)	T/C/0.69/0.64	*SYCP2L/MAK*	0.20 (0.03)	2E−12	0.16 (0.03)	1E−07	4E−01
6p21.33	rs28474889 (0.41)	C/T/0.73/0.80	*MSH5/HLA*	0.24 (0.03)	5E−16	0.16 (0.04)	4E−06	1E−01
8p12	rs28807105 (0.30)	G/A/0.30/0.22	*EIF4EBP1*	0.16 (0.03)	2E−08	0.43 (0.04)	6E−32	6E−09
12q13.3	rs2277339 (1)	T/G/0.80/0.89	*PRIM1/TAC3*	0.29 (0.03)	7E−20	0.33 (0.05)	1E−11	5E−01
17q21.31	rs8176071 (0.98)	GTGT/G/0.34/0.33	*BRCA1*	0.16 (0.03)	1E−08	0.15 (0.03)	3E−06	8E−01
Menarche								
1q23.3	rs78408536 (0.60)	CT/C/0.72/0.86	*RXRG*	0.05 (0.01)	5E−08	0.07 (0.01)	6E−09	4E−01
2q33.1	rs35020808 (0.88)	GT/G/0.50/0.68	*SATB2*	0.06 (0.01)	5E−11	0.05 (0.01)	7E−07	2E−01
6q16.3	rs11285463 (1)	AT/A/0.29/0.45	*LIN28B*	0.08 (0.01)	3E−16	0.11 (0.01)	5E−42	2E−02
8q21.11	rs7821604 (1)	C/G/0.51/0.85	*ZFHX4*	0.05 (0.01)	2E−08	0.03 (0.01)	7E−03	2E−01
9q31.2	rs1516883 (0.91)	G/A/0.53/0.69	*TMEM38B*	0.06 (0.01)	4E−12	0.12 (0.01)	5E−39	2E−05
11q24.1	rs144048300 (0.26)	T/A/0.16/0.10	*C11orf63*	0.07 (0.01)	1E−08	0.05 (0.01)	2E−04	4E−01
14q32.2	rs142252570 (1)	CTAAT/C/0.82/0.95	–	0.07 (0.01)	2E−09	0.09 (0.02)	1E−06	3E−01

Associations with menarche/menopause were analysed in 67,029/43,861 Japanese (BBJ) and 73,397/32,545 European (UK Biobank) women

^a^*r*^2^ between Japanese and European reported lead SNP calculated in Japanese

^b^Effect allele/Other allele/Effect allele frequency (EAF) in Japanese/Europeans

For menarche, 10 independent signals reached genome-wide significance, two of which are novel loci, and a third represents a novel Japanese-specific signal in a known European locus (*r*^2^~0) near *PTPRD* (Table 1; Fig. 1). To evaluate the effect of BMI as a potential confounder, we tested the association of all significant loci with additional adjustment of BMI at recruitment. There was no appreciable attenuation of test statistic observed at any of the loci (Supplementary Data 4), indicating that these variants exert their effects through pathways independent of adiposity.

Of the 12 novel signals for the two traits, 5 showed larger effect sizes in Japanese than in Europeans (*P*_heterogeneity_ < 0.004; i.e. = 0.05/12; Table 1). A further three signals were likely not identified in previous GWAS due to markedly lower allele frequencies in Europeans: *EIF4E* (rs199646819, minor allele frequency (MAF) in Japanese vs. Europeans: 39 vs. 2%), *NKX2-1* (rs2076751: 25 vs. 7%) and *THOC1* (rs77001758: 40 vs. 0.1%). In a meta-analysis allowing for trans-ethnic heterogeneity, 10 of the 12 signals remained genome-wide significant when combined with European data (Supplementary Data 4).

Four novel signals were highly correlated (*r*^2^ > 0.7) with missense variants, implicating the genes *GNRH1*, *HMCES*, *ZCCHC2* and *ZNF518A* in the regulation of menopause timing. Notably, rs6185 (Trp16Ser) in *GNRH1* is exactly the same lead SIFT-predicted deleterious missense variant recently reported for age at menarche in Europeans^5^. In our Japanese sample, the rs6185 G-allele was associated with later menopause (beta = 0.19 years per allele, *P* = 3 × 10^−13^) and later menarche (*P* = 3.5 × 10^−5^), but it reportedly has no effect on menopause timing in Europeans (beta = 0.03 years per allele, *P* = 0.16, *N* = 67,602). *HMCES* at 3q21.3 encodes an embryonic stem cell-specific binding protein for 5-hydroxymethylcytosine, a recently described epigenetic modification that is dynamically regulated during oocyte ageing^13^, while *ZNF518A* at 10q24.1 encodes an interaction partner of the epigenetic silencing machineries G9a/GLP and Polycomb Repressive Complex 2^14^.

### Biological processes, tissues and cell type enrichment

We next sought to systematically test which biological processes, tissues and cell types were most represented by genetic variation associated with menarche and menopause. Firstly, genome-wide pathway analyses were performed using MAGENTA (Methods). A total of 13 pathways were significant after multiple test correction, all for age at menopause. These results recapitulated previously observed associations in Europeans^5,7^, particularly highlighting DNA damage response mechanisms (Supplementary Data 5 and 6). Secondly, we identified significantly enriched cell and tissue types using two approaches – MAGMA and LDSC-SEG (Methods). Again, these results were broadly consistent with observations from larger European datasets, highlighting particular enrichment with all three components of the hypothalamic–pituitary–ovarian axis (Supplementary Table 3 and Supplementary Figure 3). Finally, we integrated both gene expression and methylation data at the level of individual loci. Using human RNA-seq data from the GTEx project, we focused enrichment analyses on the three components of the HPG-axis (Supplementary Data 7 and 8). This highlighted one gene for menarche (*LIN28B*) and three for menopause (*NRB2*, *LY6G5C* and *BAG6*). Methylation data were only available in whole blood, where integration identified 101 CpG sites significantly associated with menarche or menopause (Supplementary Data 9).

### Genetic associations with early or late menarche timing

As was recently shown in Europeans^5^, we tested in our Japanese GWAS sample whether variants associated with continuous age at menarche have disproportionately larger effects on early vs. late puberty timing in females. The approximate earliest (9–12 years inclusive: *N* = 15,709) and latest (16–20 years: *N* = 10,875) strata of age at menarche in Japanese were each compared to the same reference group (14 years: *N* = 14,557). Consistent with findings in Europeans^5^, in Japanese more variants had larger effects on early than on late menarche timing (Supplementary Data 11, 55.7%, 191/343, binomial *P* = 0.02).

**Fig. 2:**
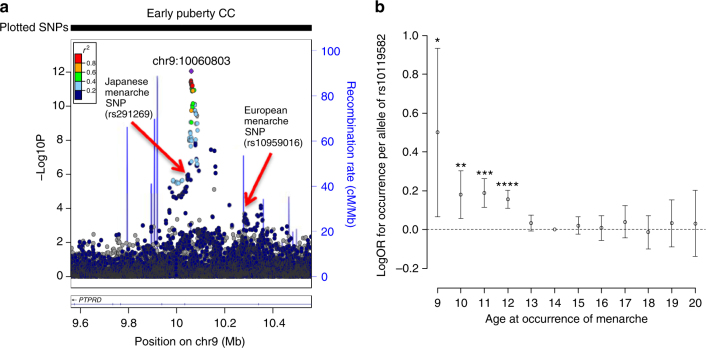
Disproportionate effects on early vs. late puberty for rs10119582 at the *PTPRD* locus. **a** Regional association plot for the early puberty model in Japanese. **b** Effect of rs10119582 at the *PTPRD* locus on risk of specific ages at menarche. Each point represents the natural log of the odds ratio per +1 ‘T’ allele at rs10119582 for being in that menarche age category compared to the reference group (menarche at age 14 years). Error bars represent the 95% confidence intervals around this estimate. *P = 0.02, **P = 0.004, ***P = 6.4 × 10^−7^, ****P = 1.3 × 10^−10^

We then tested variants genome-wide for early or late menarche timing in Japanese. We identified just one signal at *P* < 5 × 10^−8^, rs10119582 near *PTPRD* associated with early menarche timing (C-allele: OR 1.17 [1.12–1.23], *P* = 8.9 × 10^−13^), which was partially correlated with the novel signal for continuous age at menarche in Japanese (*r*^2^ = 0.31 with rs291269) and the known European menarche signal at this region (*r*^2^ = 0.11 with rs10959016) (Fig. 2a). Re-analysis of the early menarche model for rs10119582 conditioned on those two other SNPs showed no appreciable change to the magnitude of effect (Supplementary Data 11, beta was attenuated by 13%, conditional *P* = 3.3 × 10^−7^). An examination of rs10119582 by each completed whole year of menarche showed that its effects were confined to those ages earlier than the median (age 14), without any apparent effect on menarche timing when older than the median age (Fig. 2b).

### Receptor-like protein tyrosine phosphatase genes

Receptor-like protein tyrosine phosphatases (PTPRs) are a family of 20 cell-surface proteins with intracellular phosphotyrosine phosphatase activity^15^. In addition to the 2 Japanese-specific signals at *PTPRD*, for continuous and early age at menarche, in Europeans 6 further independent signals have been described for menarche (in/near: *PTPRD*, *PTPRF*, *PTPRJ*, *PTPRK*, *PTPRS* and *PTPRZ1*) with 2 others just short of genome-wide significance (*P* < 6 × 10^−8^, in/near: *PTPRG* and *PTPRN2*). In combination, this gene family is enriched for variant associations with age at menarche (MAGENTA pathway enrichment *P*_Euro_ = 7 × 10^−3^).

**Fig. 3:**
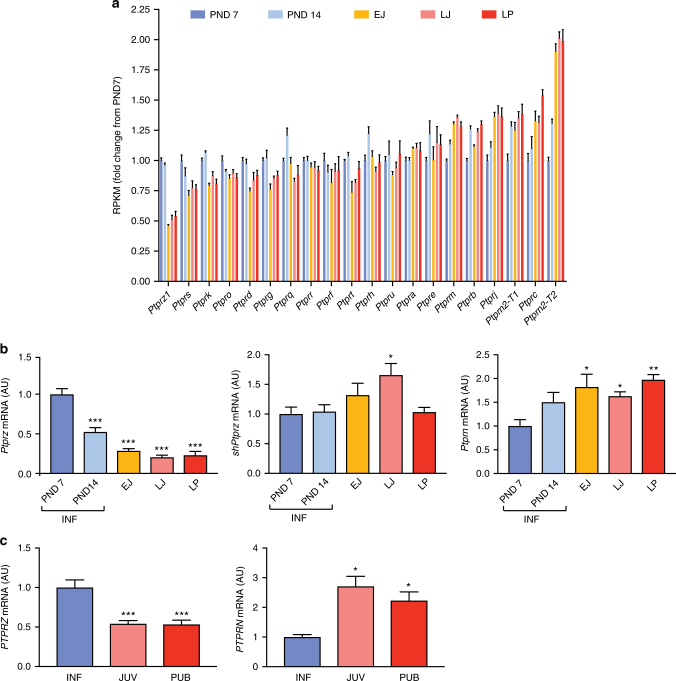
Changes in hypothalamic *PTPR* gene expression during prepubertal development of female rats and rhesus monkeys. **a** *PTPR* mRNA levels in the medial basal hypothalamus (MBH) of pre– and peripubertal female rats assessed by massively parallel sequencing (*n* = 4 biological replicates per developmental stage). **b** mRNA abundance of two selected *Ptpr* genes (*Ptprz and Ptprn*) in the MBH of pre- and peripubertal female rats assessed by qPCR. Both the long and short forms of *Ptprz* are shown. *P < 0.05; **P < 0.01 and ***P < 0.001 vs. PND 7, one-way ANOVA followed by Student-Newman-Keuls (SNK) multiple comparison test, *n* = 6–8 rats per group. INF = infantile period (PND7 and PND14); EJ = early juvenile (PND21); LJ = late juvenile (PND28); LP = late puberty (day of the first preovulatory surge of gonadotropins). **c** *PTPRZ* and *PTPRN* mRNA levels in the MBH of pre– and peripubertal female rhesus monkeys. *P < 0.05 and ***P < 0.001 vs. infantile (INF) group, one-way ANOVA followed by SNK multiple comparison test, *n* = 4–8 monkeys per group. INF = infantile (1–6 months of age); JUV = juvenile (6–19 months of age); PUB = pubertal (24–36 months of age; defined by the presence of elevated plasma LH levels). Bars represents the mean ± s.e.m

To explore the role of the PTPR gene family in the physiological regulation of puberty timing, we examined changes in gene expression, assessed by RNA-sequencing, in rat medial basal hypothalamus at 5 time points from infancy (postnatal days, PND7 and 14), through juvenile development (EJ, early juvenile PND21; LJ, late juvenile PND28), to the peripubertal period (LP, day of the preovulatory surge of gonadotropins). In false discovery rate-corrected analyses, 13 of the 20 PTPR genes examined were differentially expressed over time: 6 genes were upregulated (*PTPRB*, *PTPRC*, *PTPRJ*, *PTPRM*, *PTPRN* and *PTPRN2*) (all FDR < 0.014), and 7 genes were downregulated (*PTPRD*, *PTPRG*, *PTPRK*, *PTPRO*, *PTPRS*, *PTPRT* and *PTPRZ1*) (Fig. 3a, b; Supplementary Table 4). Additional examination of medial basal hypothalamus expression in a primate model, assessed by quantitative PCR of selected PTPR genes, showed similar time trends in expression as seen in rat hypothalamus: expression of *PTPRN* was up-regulated and *PTPRZ* was downregulated from infancy through puberty (Fig. 3c).

## Discussion

Our study represents a large non-European ancestry genomic analysis for reproductive ageing, identifying genome-wide significant loci for ages at menarche and menopause in an East Asian population. While the overall heritability estimates were lower in Japanese than in European ancestry populations, we present to our knowledge the first evidence for a secular trend in the heritability for any non-behavioural trait. Secular trends towards earlier age at menarche are widely reported^16^ and are accompanied by secular declines in its variance^17^ – our findings may suggest that such changes may be explained by declining population variability in exposure to environmental factors that delay puberty, such as childhood undernutrition^18^. Despite these population differences in heritability, our findings support a largely shared genetic architecture of reproductive ageing, notably with the replication at genome-wide significance in Japanese of 14 known European signals for menarche or menopause (Table 1). However, both effect allele frequencies and effect estimates varied considerably between populations, likely due to a combination of differential genetic drift, selection, recombination and possibly also environmental setting, resulting in substantial heterogeneity in genetic associations between the population groups and reinforcing the need to appropriately model such trans-ethnic differences in meta-analyses.

Such differences in genetic architecture underpin the value of studying genetic associations in diverse population groups to identify novel signals. Hence, even in a Japanese dataset considerably smaller than the largest reported European meta-analysis^5^, we identified 10 novel loci for ages at menarche or menopause, and these findings implicated novel genes and pathways as involved in human reproductive ageing. In addition to *HMCES* and *ZNF518A* described above, at the novel menarche locus at 14q13.3, the nearest gene *NKX2-1*, encodes a homeodomain gene that is required for basal forebrain morphogenesis and also remains active in the adult nonhuman primate hypothalamus, where its ablation in mice results in delayed puberty, reduced reproductive capacity, and a short reproductive span^19^, but, until now, has not been implicated in human reproductive function. At the novel menopause locus at 4q23, the nearest gene *EIF4E* encodes a key translation initiation factor; EIF4E is the target of the inhibitory binding protein encoded by *EIF4EBP1*, which is near to a known European ancestry menopause locus^7^, while a rare deleterious stop mutation in *EIF4ENIF1*, which encodes a nucleocytoplasmic shuttle protein for EIF4E, segregates with primary ovarian insufficiency (menopause at ~30 years old) in a large kindred^20^. Other novel menopause loci implicate: the evolutionarily conserved maternal-effect gene *ZAR1*, which encodes an oocyte-specific protein that is critical for oocyte-to-embryo transition^21^; *H1FX*, which encodes a member of the histone H1 family; and *RAD21*, a gene involved in chromatid cohesion during mitosis and the repair of DNA double-strand breaks, and mutated in two children with Cornelia de Lange syndrome-4, a complex disorder with cellular characteristics of decreased chromatid separation, increased aneuploidy and defective DNA repair^22^. Ultimately, functional work in various cellular and animal models will be required to explore all of our highlighted genomic associations in more detail and fully elucidate the pathway linking DNA sequence variation to phenotypic consequence.

Our identification of a deleterious variant rs6185 (Trp16Ser) in *GNRH1*, a known signal for age at menarche, as a novel locus for menopause timing suggests an unexpected primary role of hypothalamo-pituitary GnRH signalling in the onset of menopause. Typically, menopause is characterized by ovarian failure and accompanied by a secondary (presumed passive) rise in GnRH-driven gonadotropin secretion. Our finding that the rs6185 G-allele, which delays menarche, also delays menopause is consistent with similar reported effects of alleles near *FSHB*^23,24^, and together suggest that lower levels of gonadotropin secretion may extend reproductive lifespan. Alternatively, GnRH receptor mRNA has been recognized in human ovary, where it may mediate reported autocrine/paracrine actions of GnRH to induce apoptosis of ovarian granulosa cells^25^. Interestingly, despite consistent associations between rs6185 and menarche timing in both Japanese and Europeans, which argues against population differences in LD with an unseen causal variant, we saw a fivefold greater effect of rs6185 on menopause timing in Japanese than Europeans, a difference that suggests some yet identified strong environmental modification. Future studies, both within populations and across populations, should seek to comprehensively assess environmental modifiers at the level of individual loci and overall heritability.

Finally, we provide multiple sources of evidence in support of a role for receptor-like protein tyrosine phosphatases (PTPRs) in the regulation of puberty timing, spread across most of the 8 PTPR sub-types (summarized in Table 2). The PTPRs are involved in important developmental processes, including the formation of the nervous system by controlling axon growth and guidance^15^. Inactivation of *Ptprs* in the mouse is reported to result in hyposmia and structural defects in the hypothalamus and pituitary^26^. Directly relevant to the regulation of pubertal timing is the observation that in prepubertal female mice a short isoform of PTPRZ1 (also known as RPTPβ) expressed in astrocytes interacts with the glycosylphosphatidyl inositol-anchored protein contactin expressed in GnRH neurons^27^. Because contactin is particularly abundant in GnRH nerve terminals, it has been postulated that GnRH neuron-astrocyte communication is in part mediated by RPTPβ-contactin interactions during female reproductive development^27^. Collectively, our human genomic data in European and Japanese datasets, in addition to these animal models suggest that PTPRs are involved in the regulation of puberty timing. Additional functional work, particularly linking our identified sequence variation to *PTPR* function in humans, is required to help further understand the role this gene family plays in pubertal regulation.

**Table 2:** Summary of Receptor-like protein tyrosine phosphatase (PTPR) genes implicated in the regulation of puberty through Japanese and European genome-wide association studies (GWAS) for age at menarche, and pre- and peri-pubertal changes in hypothalamic gene expression

PTPR sub-type	Genes^a^	GWAS	Expression-up	Expression-down
I	C		C	
IIA	D,F,S	D,F,S		D,S
IIB	K,M,U,T	K	M	K,T
III	B,H,J,O,Q	J	B,J	O
IV	A,E			
V	G,Z1	G,Z1		G,Z1
VI	R			
VII	N,N2	N2	N,N2	

^a^Gene names are abbreviated from PTPR*, where * is a letter (or letter/number)

## Methods

### BBJ participants and phenotyping

All participants were recruited from Biobank Japan (BBJ), which is a patient-oriented biobank established in Japan^11^. The study protocol was approved by the research ethics committees of the Institute of Medical Science, the University of Tokyo, the RIKEN Yokohama institute and cooperating hospitals. Written informed consent was obtained from all participants. Approximately 200,000 patients diagnosed with any of the 47 targeted common diseases were enroled in BBJ between 2003 and 2008, and DNA, serum and clinical information were collected from each patient via 66 hospitals across Japan. The current analysis was based on 69,616 female participants who provided information on either age at menarche or menopause and had genotype data available. Ages at menarche or menopause were recalled to the nearest whole completed year at baseline and at multiple follow-up visits. Participants were excluded based on the following conditions: (i) missing age at menarche or menopause; (ii) missing age at recruitment; (iii) maximum difference in the recalled ages at menarche or menopause collected on multiple visits >5 years; (iv) age at recruitment was younger than reported age at menarche or menopause; (v) missing birth year (for analyses of age at menarche); (vi) age at menarche <9 or >20 years; (vii) age at menopause <40 or >60 years and (vii) patients with medical history of hysterectomy, ovariectomy, radiation, chemotherapy and hormone replacement treatment (for analyses of age at menopause). Where age at menarche or menopause was reported at multiple visits, mean values for each were calculated. In total, 67,029 participants with age at menarche and 43,861 with age at menopause were included in the quantitative trait analyses. Age at menarche was also stratified into ‘early’ (ages 9–12 years inclusive, *N* = 15,709) and ‘late’ (ages 16–20 years inclusive, *N* = 10,875), and each of these two groups was compared to the same median reference group (age 14, *N* = 14,557).

### Genotype QC imputation and discovery GWAS analysis

BBJ participants had DNA genotyped on more than 950,000 variants using either (a) a combination of Illumina Human OmniExpress BeadChip and Infinium HumanExome BeadChip or (b) Infinium OmniExpressExome BeadChip alone. Variants overlapping across these two sets of genotyping arrays were extracted. Variants were then excluded according to the following criteria: (i) monomorphic in any chip; (ii) call rate <99%; (iii) minor allele count in heterozygotes <5; (iv) Hardy-Weinberg Equilibrium *P*-value <1 × 10^−6^ in any chip. For sample quality control, we excluded samples with (i) call rate <98%; (ii) discordant phenotypic and genotypic sex; (iii) excess heterozygosity; (iv) cryptic relatedness assessed by pi_hat measurement (>0.2) for identity by descent; or if (v) not from mainland Japan identified by principal component analysis using all samples from the 1000 Genomes Project^12^. After quality control, 532,488 autosomal variants were phased using Eagle2^28^ and subsequently imputed up to the reference panel from the 1000 Genomes Project Phase 3 using minimac3^29^.

Variants with good imputation quality (minimac rsq >0.3)^30^ were tested for associations with two quantitative traits, age at menarche and age at menopause, and two dichotomous traits, early menarche and late menarche, assuming additive allelic effects. Associations with ages at menarche and menopause were tested in linear regression models using mach2qtl^31^. Associations with early and late menarche (both vs. the median group), or each age year of age at menarche (age 9, 10, 11, 12, 13, 15, 16, 17, 18, 19 and 20) vs. the same median (age 14) group), were tested in logistic regression models using mach2dat^31^. In each model, ten principal components were included to adjust for cryptic population structure, in addition to birth year as a covariate.

Variance explained by genetic variants in the current study were estimated using the Restricted Estimate Maximum Likelihood (REML) method implemented in BOLT-LMM^32^. We tested different SNP sets: (i) Directly genotyped variants within 250 kb up- or down-stream of the previously reported European lead variants^5,7^, and (ii) all directly genotyped variants which passed quality control. The same methodology and default parameters were applied to both the BBJ and UK Biobank study, using ancestry-specific LD scores generated from the 1000 Genomes project. Pathway enrichment was performed on 3216 pathways defined in Gene Ontology, PANTHER, KEGG and Ingenuity using MAGENTA^33^. The same approach was used to test the set of 20 PTPR family genes. All default parameters were used, and significance was based on an FDR<0.05 for the 75th enrichment centile.

### Gene expression integration

Tissue enrichment analyses were performed using MAGMA^34^ (implemented through FUMA^35^) and LDSC-SEG^36^. Software supplied default parameters were used for both approaches, and East-Asian specific LD scores and LD information were used where appropriate. Tissue enrichment was performed on tissues from the V6p GTEx release. Individual locus expression and methylation data were incorporated using Summary Mendelian Randomization (SMR)^37^. We used a subset of 1344 unrelated East Asian individuals (based on principle component analysis and self-reported ancestry) from the UK Biobank study (500 K HRC release) as an LD reference panel for SMR analysis. Analysis was performed using the pre-supplied McRae et al. methylation data and V7 GTEx gene expression data (http://cnsgenomics.com/software/smr/#Download). All analyses used a Bonferroni correction based on the number of tests performed (i.e 0.05/N genes).

### Effect estimate comparisons with European-ancestry samples

Known menarche and menopause European loci were defined as those discovered in the two largest reported GWAS meta-analyses to date^5,7^. As effect estimates reported in discovery meta-analyses are potentially inflated due to the ‘winners curse’ phenomenon, we derived more robust European effect estimates in independent samples from the UK Biobank study^38^. For menarche, this required us to restrict the number of known European loci to the largest discovery meta-analysis prior to inclusion of UK Biobank^5^. A total of 73,397 women with genotype and age at menarche were available from UK Biobank^5^. Age of natural menopause was available for 32,545 UK Biobank women, using the same inclusion/exclusion criteria applied to BBJ women. This analysis was performed using a linear mixed model implemented in BOLT^39^. Trans-ethnic meta-analysis was performed using Han and Eskin’s Random Effects model, implemented in Metasoft^40^.

### Animal samples for hypothalamic gene expression

Sprague Dawley female rats were studied at different phases of postnatal development: infantile postnatal day (PND) 7 and 14, early juvenile (EJ) PND21, late juvenile (LJ) PND28 and late puberty (LP, the day of the first preovulatory surge of gonadotropins, PND32–38. The use of rats was approved by the ONPRC Animal Care and Use Committee in accordance with the NIH guidelines for the use of animals in research. The animals were obtained from Charles River Laboratories international, Inc. (Hollister, CA), and were housed in a room with controlled photoperiod (12/12 h light/dark cycle) and temperature (23–25 °C). They were allowed ad libitum access to pelleted rat chow and water. The medial basal hypothalamus (MBH) of female rats was collected at various postnatal ages as described^41^. The tissues were frozen on dry ice and stored at −85 °C before RNA extraction.

Female rhesus monkey (*Macaca mulatta*) hypothalamic tissue samples were obtained through the Oregon National Primate Research Center (ONPRC) Tissue Distribution Program for the studies of infantile-pubertal (INF-PUB) transitions. The animals were classified into different stages of development based on their age and pubertal stages, following the recommendation reported by Watanabe and Terasawa^42^. These developmental stages were: (1) Infantile (3–6 months of age, characterized by relatively elevated plasma LH levels, and immature aspect of the external genitalia; (2) Juvenile (between 6–20 months of age), characterized by low plasma LH levels, and an absence of external signs of puberty; and (3) pubertal (24–44 months of age), characterized by increased plasma LH levels, initiation of menstruation, and appearance of sex-skin colour changes, without evidence of ovulation. The brain was removed from the cranium and the MBH was dissected as previously reported^43^. The tissues fragments were then snap-frozen in liquid nitrogen and stored at −80 °C.

### RNA extraction and reverse transcription PCR

Total RNA was extracted from the MBH tissues of female rats and rhesus monkeys at different developmental stages using the RNeasy mini kit (Qiagen, Valencia, CA) following the manufacturer’s protocol. DNA contamination was removed from the RNA samples by on-column digestion with DNAse using the Qiagen RNase-free DNase kit (Qiagen, Valencia, CA) according to the manufacturer’s instructions. RNA concentrations were determined by spectrophotometric trace (Nanodrop, ThermoScientific, Wilmington, DE). Five-hundred ng of total RNA were transcribed into cDNA in a volume of 20 µl using 4 U of Omniscript reverse transcriptase (Qiagen, Valencia, CA).

Relative mRNA abundance was determined using the SYBR GreenER™ qPCR SuperMix system (Invitrogen, Carlsbad, CA). Suitable amplification primers were designed using the PrimerSelect tool of DNASTAR 14 software (Madison, WI) or the NCBI online Primer-Blast program (Supplementary Table 5). PCR reactions were performed in a volume of 10 μl containing 1 μl of diluted cDNA, 5 μl of SYBR GreenER™ qPCR SuperMix and 4 μl of primers mix (1 µM of each gene specific primer). The PCR conditions used were as follows: 5 min at 95 °C, 40 cycles of 15 s at 95 °C and 60 s at 60 °C. To confirm the formation of a single SYBR Green-labelled PCR amplicon, each PCR reaction was followed by a three-step melting curve analysis consisting of 15 s at 95 °C, 1 min at 60 °C, ramping up to 95 °C at 0.5 °C per sec, detecting every 0.5 s and finishing for 15 s at 95 °C, as recommended by the manufacturer. All qPCR reactions were performed using a QuantStudio 12 K Real-Time PCR system (Thermo Fisher, Waltham, MA); threshold cycles (CTs) were detected by QuantStudio 12 K Flex software (Thermo Fisher, Waltham, MA). Relative standard curves were constructed as reported^41,43^.

### Next generation RNA sequencing and analysis

RNA extracted from the MBH of prepubertal female rats was subjected to RNA-seq following a procedure we recently described^44^. The existence of differential gene expression in the medial basal hypothalamus during pubertal development was determined by employing the edgeR^45^ analysis package. An initial trimming and adaptor removal step was carried out using Trimmomatic^46^. Removal of the Illumina adapter sequences and default filtering parameters was performed as suggested in the program’s documentation, with exception of a hard clip of the first 13 bases of the reads; this latter step was based on FastQC visualization of the read qualities before trimming, which indicated significantly lower read qualities in those bases as compared to the remainder of the read. Trimmed reads that passed the Trimmomatic selection criteria were then aligned to the rn6 build of the rat genome with Bowtie2/Tophat2^47,48^ using default parameters. Aligned reads were assigned to gene-level genomic features of the Ensembl 83 annotation set using the Rsubread featureCounts R function with countMultiMappingReads and allowMultipleOverlaps flags set to TRUE. Differential expression between time points was then analysed using the generalized linear modelling approach implemented in edgeR, based on the methods described in detail in that package’s documentation. Dispersion parameters for the regression model were estimated using sequential application of edgeR’s global and tagwise methods after normalization for library size. The design matrix used for the analysis included both indicator variables for time point and batch effects. The latter was employed to account for any variability arising from runs carried out on different dates and different flow cells; aside from time point and batch effects there were no other notable covariates of interest or potential confounders that we felt required consideration in the model. Because the model simultaneously considers the variance of all timepoints, in addition to potential batch effects, it effectively generates an integrated comparison of the entire data set and obviates the need to run repeated ANOVA tests on all ~34,000 individual genes; it also allowed us to more precisely identify differentially expressed genes based on significance values of pairwise comparisons of time points using the edgeR’s likelihood ratio test function glmLRT. In addition, we utilized the edgeR voom function to produce per-sample estimates of read counts per million for tabular visualization and to plot trends over time. Upon identification of the PTPR family as a gene set of interest, we used qRT-PCR to verify the changes detected by RNA-seq, and analysed the results by ANOVA followed by a multiple comparison test (see below).

### Quantitative PCR analysis

Statistical analysis of the qPCR results was performed using Prism7 software (Graphpad Software, La jolla, CA). Differences between groups were analysed by ONE WAY ANOVA followed by the Student-Newman-Keuls multiple comparison test for unequal replications. RNA expression data were normalized by dividing each individual value by the average of the PND7 group (in the rat) or INF group (in the monkey). Data were subjected to arc–sine transformation before statistical analysis to convert them from a binomial to a normal distribution. A *P* value of <0.05 was considered statistically significant.

### Data availability

GWAS summary statistics have been deposited in the National Bioscience Database Center (https://humandbs.biosciencedbc.jp/en/) under data set identifier hum0014.v9.Men.v1and hum0014.v9.MP.v1.

The RNA-seq data was deposited in NCBI under the accession number GSE94080.

## Electronic supplementary material

Supplementary Information

Supplementary Data 1

Supplementary Data 2

Supplementary Data 3

Supplementary Data 4

Supplementary Data 5

Supplementary Data 6

Supplementary Data 7

Supplementary Data 8

Supplementary Data 9

Supplementary Data 10

Supplementary Data 11

Description of Additional Supplementary Files
