# Supplementary material for: Elucidating the genetic architecture of reproductive ageing in the Japanese population
Source: Nat Commun. 2018 May 17;9:1977. doi: 10.1038/s41467-018-04398-z (PMC5958096; doi:10.1038/s41467-018-04398-z)

Supplementary Information

Elucidating the genetic architecture of reproductive ageing in the Japanese population

Horikoshi et al.

Supplementary Table 1	Study information for Biobank Japan and UK Biobank
Supplementary Table 2	The impact of birth year on age at menarche and heritability
Supplementary Table 3	GTEx tissue enrichment results performed using LDSC-SEG
Supplementary Table 4	Pre- and peri-pubertal changes in rat medial basal hypothalamus expression of 20 receptor-like protein tyrosine phosphatase (*PTPR*) genes
Supplementary Table 5	mRNA primer sequences used in animal model work
Supplementary Figure 1	Concordance of effect estimates / allele frequencies with available Chinese data for known European SNPs (menarche)
Supplementary Figure 2	Concordance of effect estimates / allele frequencies with available Chinese data for known European SNPs (menopause)
Supplementary Figure 3	Tissue enrichment analysis performed using MAGMA

Supplementary Table 1. Study Information of BioBank Japan and UK Biobank

Birth year	N	Age at recruitment (mean (SD))	BMI (mean (SD))	Current/past/never smoker/unknown (N)	Ever smoker (%)	Never smoker (%)
**(a) Menarche samples in BBJ**						
<1934	18,444	77.4 (4.7)	22.9 (3.7)	978/1395/15964/107	2373 (12.9%)	15964 (86.6%)
1934-45	21,835	65.7 (3.6)	23.3 (3.7)	1899/1924/17897/115	3823 (17.5%)	17897 (82.0%)
1946-65	19,703	51.5 (5.8)	23.1 (4.0)	3480/2323/13759/141	5803 (29.5%)	13759 (69.8%)
>1965	7,048	31.8 (5.9)	21.9 (4.1)	1631/723/4652/41	2354 (33.4%)	4652 (66.0%)
All	67,029	61.2 (14.9)	23.0 (3.9)	7988/6365/52272/404	14953 (22.3%)	52272 (78.0%)
**(b) Menarche samples in UK Biobank**						
1934-45	63,839	65.8 (2.1)	27.3 (4.8)	4111/23558/36143/27	27669 (43.3%)	36143 (56.6%)
1946-71	174,525	53.2 (6.5)	26.9 (5.3)	17150/53670/103618/87	70820 (40.5%)	103618 (59.4%)
All	238,364	56.6 (7.9)	27.1 (5.2)	21261/77228/139761/114	98489 (41.3%)	139761 (58.6%)
**(c) Menopause samples in BBJ**						
All	43,861	68.1 (9.2)	23.3 (3.9)	3979/3728/35912/242	7707 (17.7%)	35912 (81.9%)
**(d) Menopause samples in UK Biobank**						
All	108,817	59.6 (5.6)	26.9 (5.0)	8709/36208/63854/46	44917 (41.3%)	63854 (58.7%)

SD, standard deviation; BMI, body mass index.

Supplementary Table 2. The impact of birth year on age at menarche and heritability

Birth year	N	Median	Mean	SD	h^2g
<1934	18,444	15	15.19	1.98	14.2%
1934-45	21,835	14	14.16	1.57	14.8%
1946-65	19,703	13	12.93	1.39	17.6%
>1965	7,048	12	12.34	1.35	20.6%

SD, standard deviation.

Supplementary Table 3. GTEx tissue enrichment results performed using LDSC-SEG

	Menarche (GTEx)			Menopause (GTEx)		
Cell type	Coefficient	Coefficient_ std_error	Coefficient_ P_value	Coefficient	Coefficient_ std_error	Coefficient_ P_value
Brain_Hypothalamus	1.2E-08	3.7E-09	0.00037	4.7E-09	5.6E-09	0.203
Brain_Amygdala	1.1E-08	3.5E-09	0.00070	3.4E-09	5.3E-09	0.259
Brain_Anterior_cingulate_cortex_(BA24)	1.1E-08	3.5E-09	0.00073	1.9E-09	5.5E-09	0.369
Brain_Caudate_(basal_ganglia)	1.1E-08	3.6E-09	0.00105	1.6E-10	5.0E-09	0.487
Brain_Nucleus_accumbens_(basal_ganglia)	1.1E-08	3.7E-09	0.00150	-7.5E-10	5.2E-09	0.557
Brain_Hippocampus	1.0E-08	3.5E-09	0.00151	4.9E-09	5.3E-09	0.177
Brain_Putamen_(basal_ganglia)	1.1E-08	3.8E-09	0.00197	-6.4E-10	5.1E-09	0.550
Brain_Cortex	1.1E-08	3.7E-09	0.00212	7.8E-10	5.7E-09	0.445
Brain_Substantia_nigra	1.0E-08	3.6E-09	0.00270	-2.1E-09	5.3E-09	0.653
Brain_Frontal_Cortex_(BA9)	9.4E-09	3.6E-09	0.00394	-1.8E-10	5.6E-09	0.513
Brain_Cerebellar_Hemisphere	9.6E-09	3.7E-09	0.00463	-2.1E-09	5.5E-09	0.645
Fallopian_Tube	7.9E-09	4.0E-09	0.02374	9.6E-09	6.4E-09	0.067
Adrenal_Gland	7.2E-09	4.3E-09	0.04873	7.5E-09	7.3E-09	0.154
Brain_Cerebellum	6.1E-09	3.8E-09	0.05163	-4.3E-09	5.7E-09	0.774
Pituitary	6.4E-09	4.4E-09	0.07210	-1.9E-09	7.1E-09	0.608
Muscle_Skeletal	6.1E-09	4.3E-09	0.07946	3.3E-09	7.2E-09	0.323
Cervix_Ectocervix	6.1E-09	4.6E-09	0.09308	3.5E-09	6.9E-09	0.308
Brain_Spinal_cord_(cervical_c-1)	4.7E-09	3.9E-09	0.11343	-2.3E-09	5.5E-09	0.662
Thyroid	4.0E-09	3.6E-09	0.13139	5.1E-09	6.6E-09	0.221
Prostate	4.5E-09	4.3E-09	0.15081	-2.6E-09	7.5E-09	0.637
Vagina	3.6E-09	3.9E-09	0.17567	5.5E-09	6.0E-09	0.181
Heart_Left_Ventricle	2.5E-09	3.7E-09	0.24679	1.3E-08	6.5E-09	0.027
Esophagus_Muscularis	2.1E-09	3.6E-09	0.28330	6.6E-09	6.3E-09	0.148
Bladder	2.2E-09	3.8E-09	0.28436	1.3E-08	6.7E-09	0.024
Esophagus_Mucosa	1.1E-09	3.6E-09	0.37734	-1.2E-08	7.2E-09	0.955
Colon_Sigmoid	8.8E-10	3.9E-09	0.41148	1.8E-08	6.8E-09	0.005
Cervix_Endocervix	7.0E-10	4.2E-09	0.43333	8.2E-09	6.8E-09	0.114
Minor_Salivary_Gland	5.9E-10	3.8E-09	0.43767	4.5E-09	6.6E-09	0.245
Colon_Transverse	2.5E-10	4.8E-09	0.47878	2.0E-08	9.2E-09	0.017
Cells_Transformed_fibroblasts	-2.4E-10	4.0E-09	0.52356	-3.5E-09	7.1E-09	0.688
Adipose_Subcutaneous	-2.3E-10	3.7E-09	0.52470	4.2E-09	7.1E-09	0.279
Testis	-8.2E-10	3.8E-09	0.58416	-7.2E-09	6.1E-09	0.883
Heart_Atrial_Appendage	-8.5E-10	3.6E-09	0.59311	2.4E-09	6.6E-09	0.360
Artery_Coronary	-9.3E-10	3.8E-09	0.59740	5.1E-10	7.0E-09	0.471
Nerve_Tibial	-1.2E-09	3.8E-09	0.62426	3.2E-09	6.8E-09	0.322
Skin_Sun_Exposed_(Lower_leg)	-1.2E-09	3.5E-09	0.63091	5.7E-09	7.0E-09	0.209
Small_Intestine_Terminal_Ileum	-1.5E-09	4.2E-09	0.63766	1.3E-08	9.0E-09	0.081
Whole_Blood	-1.7E-09	4.5E-09	0.64322	9.2E-10	7.8E-09	0.453
Ovary	-1.6E-09	3.9E-09	0.66429	1.1E-08	6.8E-09	0.056
Breast_Mammary_Tissue	-1.6E-09	3.3E-09	0.68394	6.6E-09	7.6E-09	0.190
Stomach	-2.1E-09	3.7E-09	0.71467	3.4E-09	7.8E-09	0.333
Uterus	-2.4E-09	3.8E-09	0.73400	5.0E-09	7.0E-09	0.236
Adipose_Visceral_(Omentum)	-2.5E-09	3.7E-09	0.74907	5.9E-09	8.1E-09	0.233
Esophagus_Gastroesophageal_Junction	-2.7E-09	3.7E-09	0.76480	1.4E-08	7.2E-09	0.023
Liver	-2.9E-09	3.5E-09	0.79079	-3.9E-09	6.4E-09	0.728
Pancreas	-3.4E-09	3.9E-09	0.80489	-7.2E-09	6.4E-09	0.872
Spleen	-4.7E-09	4.6E-09	0.84278	-1.2E-09	7.5E-09	0.561
Skin_Not_Sun_Exposed_(Suprapubic)	-3.7E-09	3.7E-09	0.84644	5.4E-09	7.0E-09	0.221
Cells_EBV-transformed_lymphocytes	-4.6E-09	4.2E-09	0.86100	6.5E-09	7.7E-09	0.198
Kidney_Cortex	-4.4E-09	3.9E-09	0.87043	-1.5E-09	6.6E-09	0.590
Artery_Aorta	-4.6E-09	3.7E-09	0.89199	8.4E-09	6.6E-09	0.100
Artery_Tibial	-4.7E-09	3.7E-09	0.89844	8.5E-09	6.4E-09	0.090
Lung	-6.6E-09	3.8E-09	0.96047	1.4E-08	7.7E-09	0.038

Table shown is default output format for LDSC-SEG cell-type specific heritability analyses.
"Coefficient_P_value" represents the main association statistic used to judge significance.

Supplementary Table 4. Pre- and peri-pubertal changes in rat medial basal hypothalamus expression of 20 receptor-like protein tyrosine phosphatase (*PTPR*) genes

Gene Symbol	Expression levels					FDR	FDR
	PND7	PND14	PND21 (EJ)	PND28 (LJ)	PND32-38 (LP)	PND7-21	PND7-LP
PTPRA	1	1.00	1.10	1.11	1.08	1.2E-01	2.0E-01
PTPRB	1	1.26	1.12	1.24	1.30	3.0E-01	1.4E-02
PTPRC	1	1.10	1.33	1.31	1.54	2.6E-03	4.9E-06
PTPRD	1	0.98	0.75	0.84	0.88	1.9E-03	1.8E-01
PTPRE	1	1.22	1.01	1.15	1.13	9.1E-01	2.9E-01
PTPRF	1	0.91	0.82	0.91	0.92	2.2E-03	2.2E-01
PTPRG	1	1.02	0.76	0.85	0.88	1.1E-03	1.3E-01
PTPRH	1	1.22	1.03	0.91	0.99	9.4E-01	9.9E-01
PTPRJ	1	1.11	1.37	1.39	1.36	3.9E-09	4.4E-09
PTPRK	1	1.07	0.79	0.88	0.81	1.4E-03	3.5E-03
PTPRM	1	1.14	1.30	1.36	1.28	3.0E-04	5.7E-04
PTPRN	1	1.31	1.90	2.01	1.99	1.9E-33	2.3E-37
PTPRN2	1	1.29	1.25	1.35	1.39	1.8E-04	8.8E-08
PTPRO	1	0.91	0.85	0.90	0.86	1.0E-02	1.6E-02
PTPRQ	1	1.21	0.98	0.83	0.88	1.0E+00	6.0E-01
PTPRR	1	1.00	0.95	0.94	0.92	6.3E-01	4.6E-01
PTPRS	1	0.87	0.71	0.78	0.76	3.6E-09	1.5E-06
PTPRT	1	1.04	0.74	0.82	0.94	1.2E-02	5.8E-01
PTPRU	1	1.05	0.88	0.94	1.06	3.8E-01	7.8E-01
PTPRZ1	1	0.96	0.46	0.51	0.54	1.1E-11	3.1E-08

Expression values are mean voom-derived counts-per-million normalized to PND7. FDR: false discovery rate significance tests performed with edgeR. PND: Postnatal day, EJ: Early Juvenile, LJ: Late Juvenile, LP: Late Puberty.

Supplementary Table 5. mRNA primer sequences used in animal model work

Species	Gene	Accession number	Primers	Sequence	Amplicon (bp)
**Rat**	*Ptprz1*	NM_001170685.1	rPtprz Fw	CACAATGCAACAGTCCGGGT	170
			rPtprz Rv	TCTCAGGGTCAGCTTGCACA	
			rPtprz short Fw	CTCCATTCGCAGACGACTCA	91
			rPtprz short Rv	CAGACTCCAACCCCTCAGCTA	
	Ptprn	NM_053881.1	rPtprn Fw	TGCCTGCTGTTGTTGAGCGG	167
			rPtprn Rv	GTGACTTGTAAGAGGGGCCGT	
	Gapdh	NM_017008.4	rGapdh Fw	CTTCTTGTGCAGTGCCAGCC	103
			rGapdh Rv	CAAGAGAAGGCAGCCCTGGT	
**Rhesus Monkey**	*PTPRZ1*	NM_001278382.1	mkyPTPRZ Fw	GGACAGCACATCCCCTAGAGT	186
			mkyPTPRZ Rv	ACAGTACAGCTCTGCACTTCC	
	PTPRN	XM_015111125.1	mkyPTPRN Fw	GGCCCCTTTTGCAAGTCACC	175
			mkyPTPRN Rv	CCAGACCTGTCCCTTGGACG	
	GAPDH	NM_001195426.1	mkyGAPDH Fw	AAGGGCATCCTGGGCTACA	68
			mkyGAPDH Rv	GAAGAGTGGGTGTCGCTGTTG	

Supplementary Figure 1. Concordance of a) effect allele frequencies and b) effect estimates of European-identified Menarche SNPs in available Chinese data v.s. Japanese BBJ data

(a) **(b)**

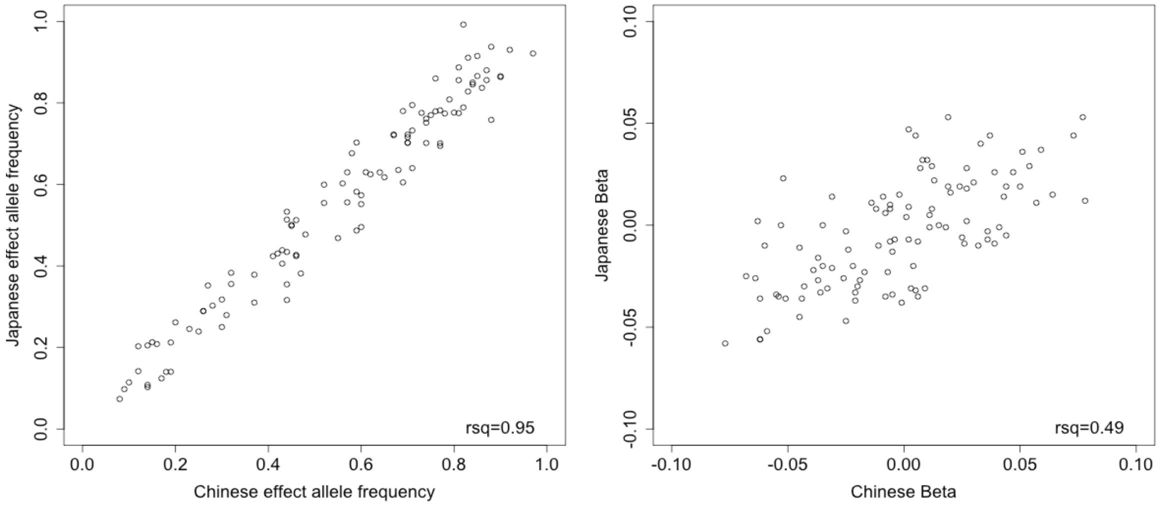

Supplementary Figure 2. Concordance of a) effect allele frequencies and b) effect estimates of European-identified Menopause SNPs in available Chinese data v.s. Japanese BBJ data

(a) **(b)**

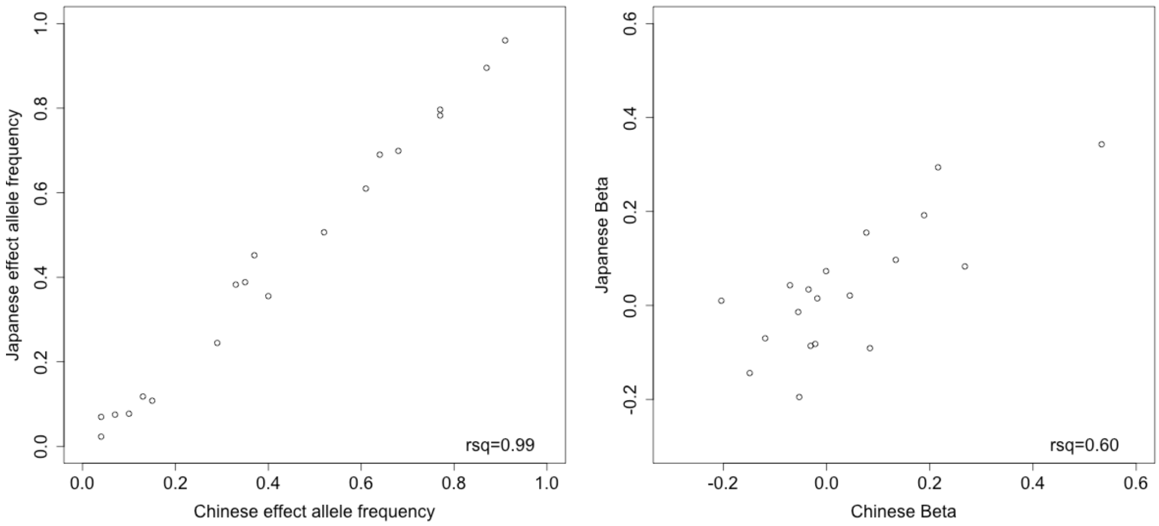

Supplementary Figure 3. Tissue enrichment analysis performed using MAGMA for a) Menarche and b) Menopause

(a)

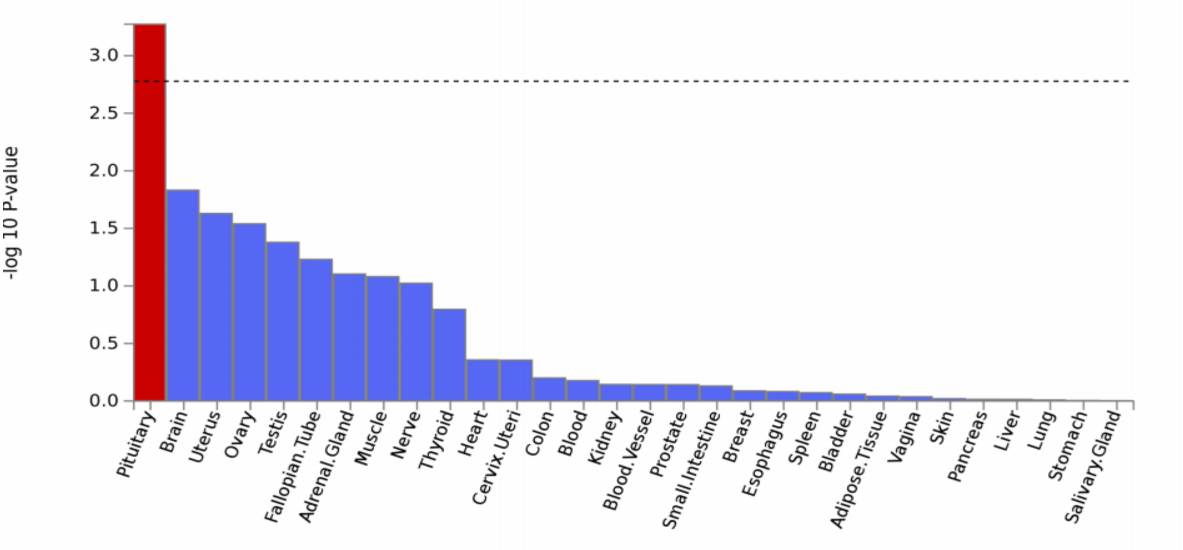

(b)

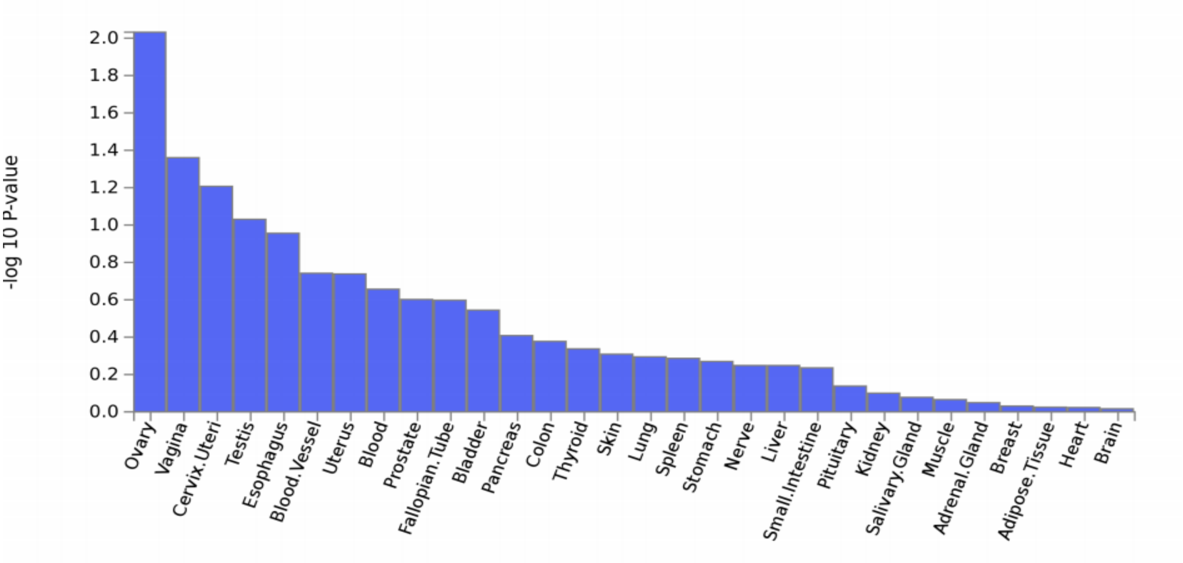

Supplement: Supplementary file 1 — Supplementary Information [file 41467_2018_4398_MOESM1_ESM.pdf]
