# Supplementary material for: Elucidating the genetic architecture of reproductive ageing in the Japanese population
Source: Nat Commun. 2018 May 17;9:1977. doi: 10.1038/s41467-018-04398-z (PMC5958096; doi:10.1038/s41467-018-04398-z)
Supplement: Supplementary file 13 — Description of Additional Supplementary Files [file 41467_2018_4398_MOESM13_ESM.pdf]

Description of Additional Supplementary Files

File Name: Supplementary Data 1
Description: A lookup of the previously reported age at menarche loci in Biobank Japan.

File Name: Supplementary Data 2
Description: A lookup of the previously reported age at menopause loci in Biobank Japan.

File Name: Supplementary Data 3
Description: A lookup of previously reported age at menarche loci, discovered independent of UK Biobank, in Biobank Japan.

File Name: Supplementary Data 4
Description: Effects of identified SNPs after adjustment for Body Mass Index.

File Name: Supplementary Data 5
Description: MAGENTA pathway results for age at menarche.

File Name: Supplementary Data 6
Description: MAGENTA pathway results for age at menopause.

File Name: Supplementary Data 7
Description: HPG-axis Summary Mendelian Randomization (SMR) results for age at menarche.

File Name: Supplementary Data 8
Description: HPG-axis Summary Mendelian Randomization (SMR) results for age at menopause.

File Name: Supplementary Data 9
Description: SMR results for whole blood methylation analyses (menarche and menopause).

File Name: Supplementary Data 10
Description: A comparison of early vs late menarche effects for known menarche loci.

File Name: Supplementary Data 11
Description: Age at menarche conditional analyses at the PTPRD gene locus.
